# Comparison of the clinical effects for different positions of the weight-bearing axis after high tibial osteotomy

**DOI:** 10.1186/s13018-023-03912-4

**Published:** 2023-06-10

**Authors:** Han Xu, Huali Tu, Tianzuo Zhao, Daofei Xu, Qinglong Yu, Long Liao, Shitian Tang, Bo Shi

**Affiliations:** Department of Orthopedics, Mianyang Central Hospital, School of Medicine, University of Electronic Science and Technology of China, No. 12, Changjia Lane, Jingzhong Street, Mianyang, 621000 China

**Keywords:** Osteoarthritis, Knee, Osteotomy, Tibia, Lower limb alignment, Clinical effects

## Abstract

**Purpose:**

To analyze the clinical effects of different positions of the weight-bearing axis (WBA) after high tibial osteotomy (HTO).

**Methods:**

The clinical data of 90 patients who underwent HTO in the Department of Orthopedics at our hospital from June 2018 to June 2021 were retrospectively analyzed. Patients were divided into groups A and B (*n* = 45 per group) according to different post-HTO WBA positions of the affected side. WBAs in both groups were at 50–60% and 62–66% of the tibial plateau, from inside to outside, respectively. American Hospital for Special Surgery Knee Score (HSS), visual analog scale (VAS) score, femorotibial angle (FTA), and medial proximal tibial angle (MPTA) were recorded and analyzed.

**Results:**

All patients were followed up with for 12 months. HSS scores increased gradually and VAS scores decreased gradually in both groups preoperatively, and at 3 months, 6 months, and 1 year postoperatively (*P* < 0.05). Compared to group A, group B had better HHS scores at 6 months and 1 year postoperatively (*P* < 0.05). There was no significant between-group difference in VAS scores at all aforementioned timepoints (*P* > 0.05). Postoperative MPTA and FTA were 89.56° ± 2.18° and 177.11° ± 2.63° in group A, and 89.07° ± 1.98° and 177.07° ± 2.36° in group B, respectively, with no significant between-group difference (*P* > 0.05).

**Conclusion:**

Patients with post-HTO WBA ranges of 50–60% and 62–66% achieved knee joint function improvement and pain relief. Half a year later, those with a WBA range of 62–66% had better knee joint function scores. However, a comparison of long-term effects warrants further investigation.

## Introduction

High tibial osteotomy (HTO) is increasingly favored by domestic doctors and patients because of the prevalence of minimally invasive procedures, knee preservation, and other conservative concepts, such as joint replacement and rejection. Its principle is to correct lower limb alignment, transfer stress to the relatively normal lateral compartment, reduce the load on the medial compartment, relieve knee joint pain, delay or prevent the continuing destruction of the medial compartment, and achieve KOA treatment or postpone knee arthroplasty [1]. However, where the stress load is transferred to the lateral compartment (i.e., where the weight-bearing axis [WBA] should pass through the tibial plateau) remains controversial. Fugisawa et al. [2] suggested that the target area of WBA should be 65–70% of the tibial platform from inside to outside, which was subsequently refined to 62.5% (range: 62–66%) [3]. Martay et al. [4] proposed 55% as the new target position of WBA. While considering possible intraoperative over- or under-correction errors, the safety zone of WBA was adjusted to 50–60% of the tibial plateau from inside to outside.

Although HTO research has progressed, the optimal WBA location remains unclear [5]. In their study of the curative effect at 10 years after HTO, Schuster et al. [6] pointed out that while HTO was a good treatment option, inadequate and over-correction of the proximal medial angle of the tibia (MPTA) would lead to poor long-term functional outcomes.

Based on these studies, the WBA of patients who received HTO in our hospital from June 2018 to June 2021 was measured and analyzed to study the clinical efficacy of different WBA positions after surgery.

## Methods

### Inclusion and exclusion criteria

The inclusion criteria were (1) KOA patients who received HTO; (2) anteromedial knee pain as the main symptom; (3) under 65 years of age; (4) normal range of motion of knee joint with flexion deformity < 10°; (5) tibial varus knee deformity, knee lateral cartilage, and meniscus function were all normal; and (6) WBA was at 50–60% and 62–66% of the tibial plateau from inside to outside.

The exclusion criteria were (1) patients with body mass index (BMI) > 30 kg/m^2^; (2) patients with knee joint multi-compartment disease; (3) knee ligament and other functional abnormalities; and 4) non-degenerative KOA.

### General information

Ninety patients were included and evenly divided into two groups (groups A and B, *n* = 45 per group) according to their WBA position on the affected side after KOA surgery. WBA positions in groups A and B were 50–60% and 62–66% of the tibial plateau from inside to outside, respectively. Group A comprised 27 men and 18 women (age: 51.87 ± 7.61 years; BMI: 24.77 ± 3.05 kg/m^2^); left knee: 18 cases, right knee: 27 cases). Group B comprised 24 men and 21 women (age: 50.13 ± 7.99 years; BMI: 23.76 ± 3.08 kg/m^2^; left knee: 19 cases, right knee: 26 cases). There were no significant differences in age, sex, BMI, or surgical side between the two groups (*P* > 0.05).

### Operative technique

All surgeries were performed by the same surgeon and team. The patients were placed in the supine position, with an upper balloon tourniquet on the affected limb, under general anesthesia, with satisfactory anesthetic disinfection, external joint puncture, and normal saline infusion. Knee arthroscopy was performed to examine the knee joint lesions and explore the articular cartilage of each compartment for HTO feasibility determination. For patients with medial compartment lesions and normal lateral compartment cartilage, HTO was performed, the synovium was cleared under arthroscopy, the meniscus was repaired, sodium hyaluronate was injected, and arthroscopy was completed.

HTO was performed with a straight incision made at the proximal end of the medial leg. The medial tibial plateau was exposed via layer-by-layer cutting. Three Kirschner needles were placed 3 cm below the platform at the top of the fibula head, and the fluoroscopic position of the C-arm X-ray machine was satisfactory. High osteotomy was performed on the tibial tubercle, a distractor was placed, and the varus angle was corrected and washed with normal saline solution. A medial tibial plateau anatomical plate (TomoFix) was inserted into the medial tibial plateau and fixed using a locking screw. When allogeneic bone was implanted into the osteotomy plane, the platform height, bone graft amount, and plate position were satisfactory under the C-arm X-ray machine. The incision was sutured layer by layer, covered with aseptic dressing, and bandaged in a straight position, before completing the operation.

### Perioperative management

Tranexamic acid and cefuroxime were administered intravenously 30 min preoperatively to reduce bleeding and prevent infection. An open wound drainage tube was routinely placed and removed on the first postoperative day. Cefuroxime was administered to prevent postoperative infection, and enoxaparin anticoagulation and multimodal analgesia were routinely administered postoperatively. After the anesthesia wore off, the patient began an ankle pump exercise. On the first postoperative day, the patient began knee flexion, extension, and straight leg elevation exercises. On the second postoperative day, the patient underwent vascular ultrasonography and X-ray examination. After no abnormality was confirmed, the recovery routine involved encouraging the patient to move with the aid of a walker.

### Observation index

Clinical evaluation included the American Hospital for Special Surgery Knee Score (HSS) and visual analog scale (VAS) score of patients, preoperatively and at 3 months, 6 months, and 1 year postoperatively.

For imaging evaluation, the femorotibial angle (FTA) and MPTA were measured and recorded on full-length anteroposterior radiographs of both lower limbs. All data were evaluated by two researchers, and the results were averaged.

### Statistical analysis

Data were analyzed using SPSS (version 22.0; IBM, Armonk, NY, USA). HSS, VAS, MPTA, and FTA data were expressed as mean ± standard deviation. An independent sample t-test was used for pairwise comparison. One-way analysis of variance (ANOVA) was used to compare multiple groups. Data were compared using the Chi-squared test. Differences were considered statistically significant at *P* < 0.05.

## Results

**Fig. 1:**
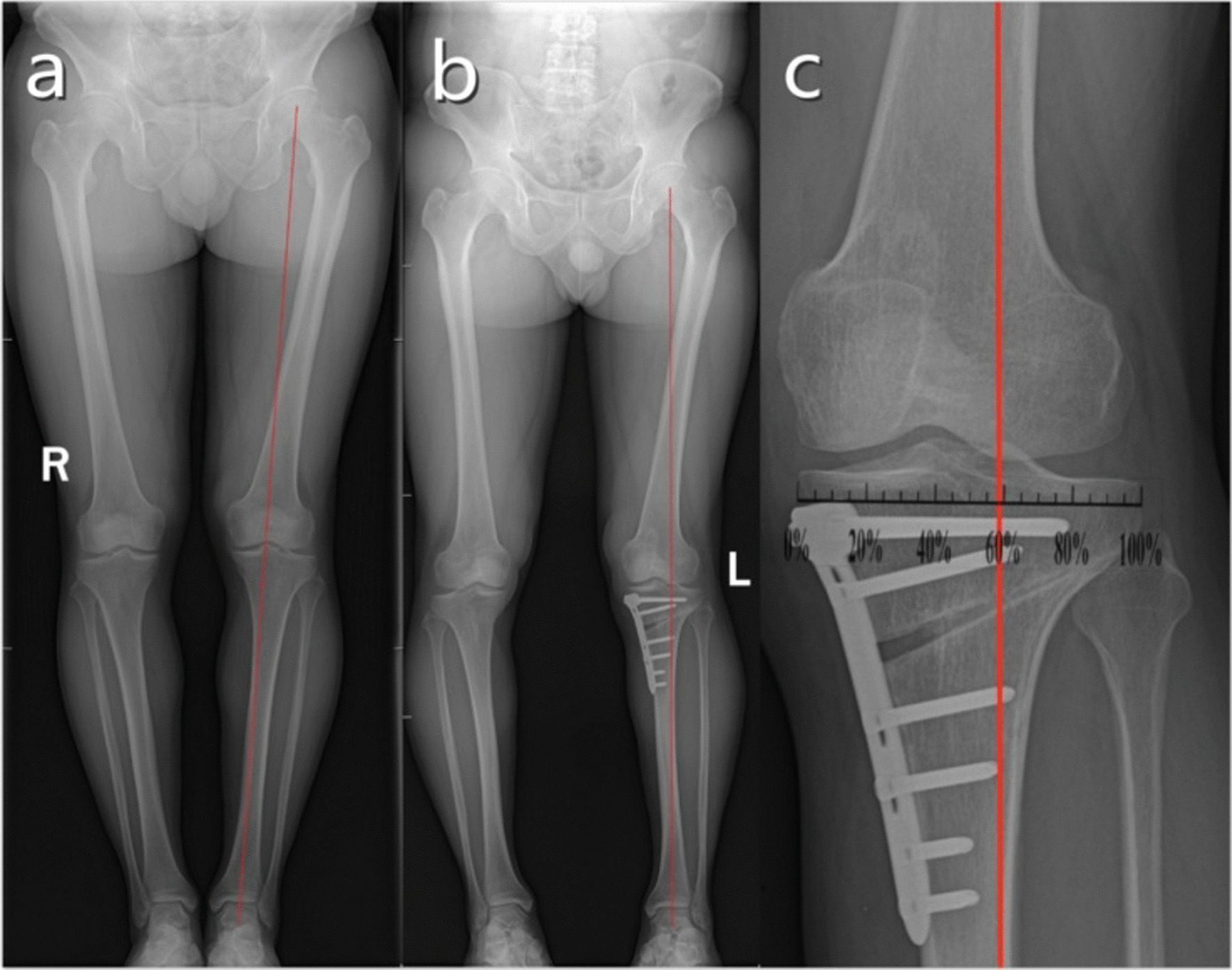
A 52-year-old male was admitted to the hospital for pain in his left knee joint for 6 months and diagnosed with knee osteoarthritis (Kellgren–Lawrence grade 3). Preoperative full-length X-rays of both lower limbs showed that the WBA of the left lower limb deviated from the medial tibial plateau (**a**). Postoperative X-ray of HTO showed that the WBA of the left lower limb deviated to the lateral tibial plateau (**b**). After correction, the WBA was located at ~ 50%–60% of the tibial plateau (**c**)

**Fig. 2:**
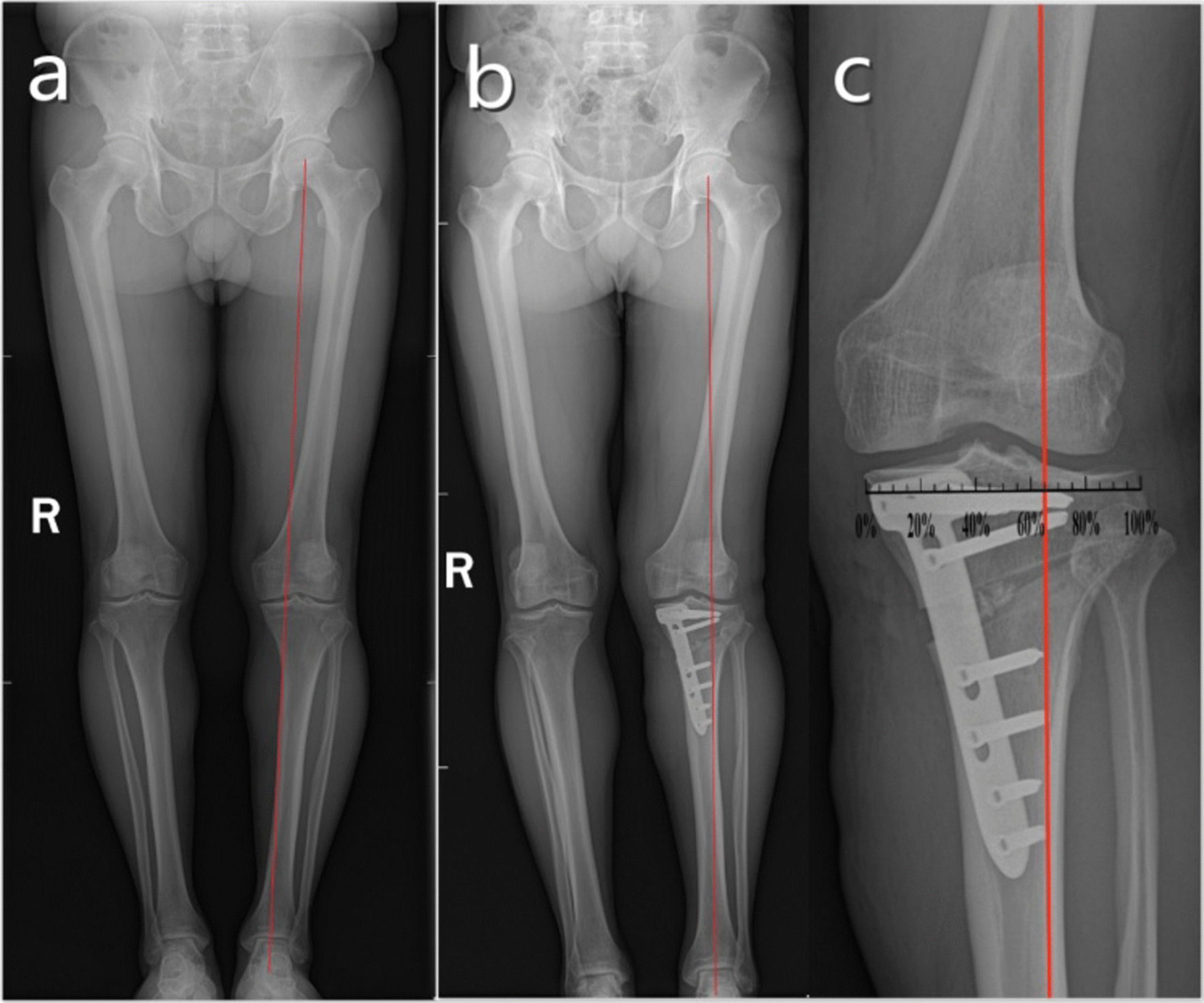
A 54-year-old male was admitted to the hospital for pain in his left knee joint for 8 months and diagnosed with knee osteoarthritis (Kellgren–Lawrence grade 3). Preoperative full-length X-rays of both lower limbs showed that the WBA of the left lower limb deviated from the medial tibial plateau (**a**). Postoperative X-ray of HTO showed that the WBA the left lower limb deviated to the lateral tibial plateau (**b**). After correction, the WBA was located at ~ 62%–66% of the tibial plateau (**c**)

All patients were followed up for with 12 months. The mean healing time of the osteotomy was 3 months. No MPTA or FTA was lost during follow-up. HSS scores increased and VAS scores decreased gradually preoperatively, and at 3 months, 6 months, and 1 year postoperatively in both groups (*P* < 0.05). Compared to group A, group B had significantly better HSS scores at 6 months and 1 year postoperatively (*P* < 0.05). There was no significant difference in VAS scores between the two groups at all aforementioned timepoints (*P* > 0.05) (Tables 1, 2). There was no significant difference in postoperative MPTA and FTA between the two groups (Table 3). Representative cases from both groups are illustrated in Figs. 1 and 2.

**Table 1:** Comparison of HSS scores between two groups

Group	Preop	3 months postop	6 months postop	1 year Postop	*F*-Value	*P*-Value
A	58.09 ± 4.99	75.07 ± 2.54	80.44 ± 3.51	85.24 ± 4.23	449.709	0.000
B	58.22 ± 4.97	75.76 ± 2.83	85.73 ± 4.24	90.17 ± 2.67	60.444	0.000
*t*-Value	− 0.124	− 1.241	− 7.788	− 5.269	–	–
*P-*Value	0.902	0.221	0.000	0.000	–	–

HSS, American Hospital for Special Surgery Knee Score; postop, postoperatively; pre-op, preoperatively

**Table 2:** Comparison of VAS scores between two groups

Group	Preop	3 months postop	6 months postop	1 year postop	*F*-Value	*P*-Value
A	2.96 ± 0.90	2.48 ± 0.51	1.13 ± 0.78	0.78 ± 0.76	470.779	0.000
B	2.93 ± 0.86	2.43 ± 0.50	1.02 ± 0.75	0.64 ± 0.68	51.206	0.000
*t*-Value	0.119	0.769	0.658	0.813	–	–
*P-*Value	0.906	0.442	0.514	0.420	–	–

VAS, visual analog scale; postop, postoperatively; preop, preoperatively

**Table 3:** Comparison of imaging indices between two groups

Groups	MPTA		FTA	
	Preoperative	Postoperative	Preoperative	Postoperative
A	84.53° ± 1.99°	89.56° ± 2.18°	183.02° ± 2.75°	177.11° ± 2.63°
B	84.16 ± 1.72°	89.07° ± 1.98°	182.80° ± 2.42°	177.07° ± 2.36°
*t*-Value	0.862	1.033	0.409	0.101
*P-*Value	0.394	0.307	0.685	0.920

FTA, femorotibial angle; MPTA, medial proximal tibial angle

## Discussion

HTO is a reliable surgical method for treating medial single compartment KOA with varus deformity in younger patients [7–9]. People are increasingly interested in the skeletal structure of deformities and its impact on outcomes and corrective strategies [10]. In the past few decades, the ideal degree of correction has been extensively researched, with suggested correction ranges ranging from neutral to extreme eversion [11, 12]. In our study, it was found that WBA after HTO surgery can achieve good knee joint function improvement and significant pain relief in patients within the most discussed range (50–60% and 62–66%). Therefore, there remains a long way to go in the study of the optimal location of WBA after correction in HTO patients.

When WBA is in the neutral position (< 50%), force line correction has little effect on intra-articular pressure change. Force line adjustment in this range can be classified as “under-correction.” The contact area and stress size of lateral and internal compartments of the knee joint were similar to those before HTO. Therefore, after correction, WBA < 50% offers limited benefits for patients. On the other hand, post-correction WBA > 65% will produce great lateral stress and pressure on the lateral compartment of the knee joint. Force line adjustment in this range can be classified as “over-correction.” Over-correction may cause damage to the lateral tissue of the knee joint, and patients may experience lateral compartment pain [13]. Therefore, Martay et al. [4] used the knee joint finite element model to study the relationship between WBA position and pressure distribution of the tibial plateau and concluded that a WBA range of 50–60% could be considered as a new safety interval. In this interval, stress and pressure in the medial compartment of the knee joint decreased, whereas those in the lateral compartment did not increase significantly. Concurrently, this safety interval would not cause damage to the lateral compartment owing to small errors in the surgical process, such as the 62.5% WBA of Fugisawa et al. [2]. Patients in group A were selected according to Martay’s criteria for our follow-up study. Postoperative results showed that the HSS scores of patients increased gradually, their VAS scores decreased gradually, their pain was relieved, and their knee joint function was significantly improved. MPTA was adjusted from 84.53° ± 1.99° preoperatively to 89.56° ± 2.18° postoperatively, and FTA was adjusted from 183.02° ± 2.75° preoperatively to 177.11° ± 2.63° postoperatively. A WBA of 62.5% (range: 62–66%), as proposed by Fugisawa et al. [2], remains the target selected by most knee surgeons. In this study, patients in group B were selected according to Fugisawa’s criteria for our follow-up study. Their pain was also reduced and their knee joint function was significantly improved. MPTA was adjusted from 84.16° ± 1.72° preoperatively to 89.07° ± 1.98° postoperatively, and FTA was adjusted from 182.80° ± 2.42° preoperatively to 177.07° ± 2.36° postoperatively. In their 2-year follow-up study of 118 patients with HTO, Sawaguchi et al. [8] showed that the average WBA after HTO increased from 23.1% to 62.4%, demonstrating that the procedure showed a reliable mechanical axis correction in subsequent weight-bearing displacement, and the knee joint function of the patients improved significantly after 2 years. Because our study relied on criteria from previous studies to select patients for discussion and focused on the discussion of the previously described WBA safety interval, patients within the WBA range of 61% were not included and corresponding studies were not conducted.

In this study, there was no significant difference in pain and imaging evaluation between groups A (WBA: 50–60%) and B (WBA: 62–66%) (*P* > 0.05). In terms of knee joint function score, HSS scores of group B at 6 months and 1 year postoperatively were significantly better than those of group A (*P* < 0.05). From these findings, there was no significant difference in clinical efficacy between the two groups. Similarly, Goshima et al. [14] divided their patients into two groups according to post-HTO MPTA at their institution, including the normal (MPTA < 95°) and over-correction (MPTA > 95°) groups. In the comparison of postoperative clinical and imaging results between the two groups, there was no significant difference in clinical results at the mid-term follow-up. Even if postoperative MPTA was slightly higher than 95°, this finding did not affect post-HTO clinical results. We analyzed the intraoperative process and clinical results of HTO and found that the reasons for the absence of significant difference were as follows: 1) WBA positions in groups A and B of our study were mostly close to the upper and lower limits, respectively, of their WBA ranges. 2) Our follow-up duration was not long and adverse conditions of the knee joint had not been shown. Our findings were similar to those reported by Akamatsu et al. [15], who performed arthroscopic examination on patients who took steel plates after HTO. These authors found that even if the force line of the lower limb was over-corrected, it would not affect the cartilage regeneration of the medial compartment of the knee joint and the cartilage degeneration of the lateral compartment postoperatively. The average interval from the initial operation to plate removal was 18.5 months, which was considered too short to evaluate the cartilage degeneration of the lateral compartment of the knee joint. Similarly, Schuster et al. [6] found that complete cartilage repair rates of femoral and tibial plateau sides were 40.9% and 29.4%, respectively, through the second arthroscopic examination of cartilage regeneration and the poor repair rates (repair area < 50%) were 15.2% and 21.6%, respectively. These findings revealed that regeneration or degeneration of knee articular cartilage was a long-term process with slow progression. Therefore, the comparison of clinical efficacy of different force line positions after HTO also requires long-term follow-up of the influence of knee joint cartilage status after surgery. Nakayama et al. [16] suggested that if post-HTO MPTA > 95° was expected (i.e., WBA would be over-corrected), whether HTO could be the optimal surgical option should be considered. However, Schuster et al. [6] reported that long-term functional outcomes decreased in cases with MPTA > 95°, but there was no difference in the 10-year survival rate, and the overall outcome was satisfactory. Therefore, these authors suggested that a certain degree of excessive correction was acceptable. However, the extent to which the correct position of WBA after HTO is acceptable remains unclear.

This study had some limitations. First, this study adopted a retrospective research method with a relatively small sample size, so it cannot avoid the occurrence of selection bias. Therefore, we used two researchers to strictly include and exclude cases, and strengthen follow-up to reduce the dropout rate to achieve the minimum selection bias. Second, the study only reflected short- and medium-term results; thus, further studies of long-term follow-up outcomes are needed. Third, although all our measurement data were averaged by two researchers, the bias caused by the difference in patient position and data measurement during postoperative X-ray imaging examination might have affected our findings.

In conclusion, patients with post-HTO WBA ranges of 50–60% and 62–66% could achieve good knee joint function improvement and obvious pain relief. Half a year later, those with a WBA range of 62–66% had better knee joint function scores. However, a comparison of long-term effects warrants further investigation.

## Data Availability

Not applicable.

## References

[1] Sabzevari S, Ebrahimpour A, Roudi MK, Kachooei AR (2016). High tibial osteotomy: a systematic review and current concept. Arch Bone Joint Surg.

[2] Fujisawa Y, Masuhara K, Shiomi S (1979). The effect of high tibial osteotomy on osteoarthritis of the knee. An arthroscopic study of 54 knee joints. Orthop Clin N Am.

[3] Dugdale TW, Noyes FR, Styer D (1992). Preoperative planning for high tibial osteotomy. The effect of lateral tibiofemoral separation and tibiofemoral length. Clin Orthop Related Res.

[4] Martay JL, Palmer AJ, Bangerter NK, Clare S, Monk AP, Brown CP, Price AJ (2018). A preliminary modeling investigation into the safe correction zone for high tibial osteotomy. Knee.

[5] Wright JM, Crockett HC, Slawski DP, Madsen MW, Windsor RE (2005). High tibial osteotomy. J Am Acad Orthop Surg.

[6] Schuster P, Geßlein M, Schlumberger M, Mayer P, Mayr R, Oremek D, Frank S, Schulz-Jahrsdörfer M, Richter J (2018). Ten-year results of medial open-wedge high tibial osteotomy and chondral resurfacing in severe medial osteoarthritis and varus malalignment. Am J Sports Med.

[7] Gaweda K, Tarczynska M (2021). Is the high Tibial osteotomy (HTO) still a valid method for treatment of medial unicompartmental knee osteoarthritis?. J Invest Surg.

[8] Sawaguchi T, Takeuchi R, Nakamura R, Yonekura A, Akiyama T, Kerstan M, Goldhahn S (2020). Outcome after treatment of osteoarthritis with open-wedge high-tibial osteotomy with a plate: 2-year results of a Japanese cohort study. J Orthop Surg (Hong Kong).

[9] Kuwashima U, Iwasaki K, Kurakazu I, Akasaki Y, Nakashima Y, Itoh M, Itou J, Okazaki K (2021). Effect of osteoarthritis severity on survival and clinical outcomes after high tibial osteotomy. Knee.

[10] Duivenvoorden T, van Diggele P, Reijman M, Bos PK, van Egmond J, Bierma-Zeinstra SMA, Verhaar JAN (2017). Adverse events and survival after closing- and opening-wedge high tibial osteotomy: a comparative study of 412 patients. Knee Surgery Sports Traumatol Arthrosc.

[11] Lobenhoffer P, Agneskirchner JD (2014). Umstellungsosteotomie vs. unikondyläre Prothese bei Gonarthrose [Osteotomy around the knee vs. unicondylar knee replacement]. Der Orthopade.

[12] Tsukada S, Wakui M (2017). Is overcorrection preferable for repair of degenerated articular cartilage after open-wedge high tibial osteotomy?. Knee Surgery, Sports Traumatol Arthrosc.

[13] Hernigou P, Medevielle D, Debeyre J, Goutallier D (1987). Proximal tibial osteotomy for osteoarthritis with varus deformity. A ten to thirteen-year follow-up study. J Bone Joint Surg.

[14] Goshima K, Sawaguchi T, Shigemoto K, Iwai S, Fujita K, Yamamuro Y (2019). Comparison of clinical and radiologic outcomes between normal and overcorrected medial proximal tibial angle groups after open-wedge high tibial osteotomy. Arthrosc J Arthrosc Related Surg.

[15] Akamatsu Y, Kumagai K, Kobayashi H, Tsuji M, Saito T (2018). Effect of increased coronal inclination of the tibial plateau after opening-wedge high tibial osteotomy. Arthrosc J Arthrosc Related Surg.

[16] Nakayama H, Iseki T, Kanto R, Kambara S, Kanto M, Yoshiya S, Schröter S (2020). Physiologic knee joint alignment and orientation can be restored by the minimally invasive double level osteotomy for osteoarthritic knees with severe varus deformity. Knee Surg Sports Traumatol Arthrosc.

